# Inkjet printing-based fabrication of microscale 3D ice structures

**DOI:** 10.1038/s41378-020-00199-x

**Published:** 2020-10-19

**Authors:** Fengyi Zheng, Zhongyan Wang, Jiasheng Huang, Zhihong Li

**Affiliations:** National Key Laboratory of Science and Technology on Micro/Nano Fabrication, Institute of Microelectronics, Peking University, Beijing, 100871 China

**Keywords:** Engineering, Materials science

## Abstract

This study proposed a method for fabricating 3D microstructures of ice without a supporting material. The inkjet printing process was performed in a low humidity environment to precisely control the growth direction of the ice crystals. In the printing process, water droplets (volume = hundreds of picoliters) were deposited onto the previously formed ice structure, after which they immediately froze. Different 3D structures (maximum height = 2000 µm) could be formed by controlling the substrate temperature, ejection frequency and droplet size. The growth direction was dependent on the landing point of the droplet on the previously formed ice structure; thus, 3D structures could be created with high degrees of freedom.

## Fabricating 3-D ice microstructures with inkjet printing

Using inkjet printing technology, a method has been developed for fabricating 3-D ice microstructures, where the ice patterns possess geometries with a resolution of tens of micrometers. Recently, inkjet technology has gained attention for use in micromanufacturing, with different deposition processes being utilized to apply a material onto a substrate. Through the phase transition of water to ice, water offers many advantages as a material in this regard, though existing technologies demand expensive equipment and a supporting layer. A team headed by Zhihong Li at Peking University, China has succeeded in developing a method where inkjet printing is used to precisely control the growth of ice crystals in the fabrication process without needing a supporting layer. The authors believe their system offers considerable potential for application with biomicrofluidic devices, such as drug delivery systems.

## Introduction

In the past decade, inkjet technology has become recognized in the technical community as a highly capable tool for manufacturing, particularly micromanufacturing^1,2^. In micromanufacturing, inkjet deposition processes are used to produce a pattern of material on a substrate. Most micromanufacturing applications of inkjet technology deposit phase change liquid inks onto nonporous substrates, which solidify quickly after deposition. Examples of phase change materials in micromanufacturing applications include solders for electronic manufacturing^3,4^ and thermoplastics for free-form fabrication^5,6^. The control of spreading by solidification is a beneficial aspect of phase change materials for applications where the goal is to limit spreading and obtain the smallest spot for a given droplet size^7^.

Water is the most common substance in nature and has substantial advantages in free contamination. The phase transition from water to ice is a phenomenon that often occurs^8^. Studies have been applied to determine the macroscale mechanism and process of icing, such as freezing front propagation^9,10^, supercooling phenomenon^11,12^, ice spike phenomenon^13^, ice layer spreading along a substrate^14^ and freezing of droplets^15,16^ and bubbles^17^. Ice has also been widely used in microfabrication, such as ice templates^18^ for biomaterial systems, ice lithography^19–21^ for nanopatterning methods and ice blocks for microfluidic channels created by laser processing^22^. However, these technologies are based on removal techniques and require expensive equipment and an additional supporting layer or protection layer.

Utilizing water as a phase change material in 3D printing, we proposed an economical additive manufacturing method to fabricate ice structures by inkjet printing, which is called “ice printing”^23^. A small liquid droplet can be printed separately from the nozzle onto a cold surface, after which the liquid droplet immediately freezes into an ice crystal. Then, an increasing number of water droplets can be printed and interconnected to create a specific structural design. This ice structure can then be used in various applications under the ice point. However, in its current form, this ice printing method is limited to simple planar structures close to the substrate, such as microcapsule ice arrays for reagent presealing^24,25^ and parallel ice channels for microfluidics^26^. In this paper, we optimized the process parameters to precisely control the growth direction of ice crystals, fully realizing the concept of 3D ice printing. A variety of ice structures with different geometries, sizes, heights and overhanging structures were successfully produced. In contrast to other 3D printing methods, such as fused deposition modeling and stereolithography, the ice printing method does not need additional supporting structures or removal processes, which simplifies the geometric design and avoids the unnecessary introduction of other chemicals. Moreover, the 3D ice printing method does not require specific substrates. The ice structure can be formed on any theoretical substrate that is sufficiently cold for icing, such as glass, metal, silicon, and polymer films. Furthermore, 3D-printed ice structures can be used as soft lithography molds for complex microfluidic channels, which is difficult or even infeasible for conventional photolithography methods. In addition, 3D ice printing has the potential to create 3D porous scaffolds of metal salt nanoparticles, which precipitate from the water during the freezing process and then occupy the spaces between the ice grains.

## Results

### Printing process

**Fig. 1:**
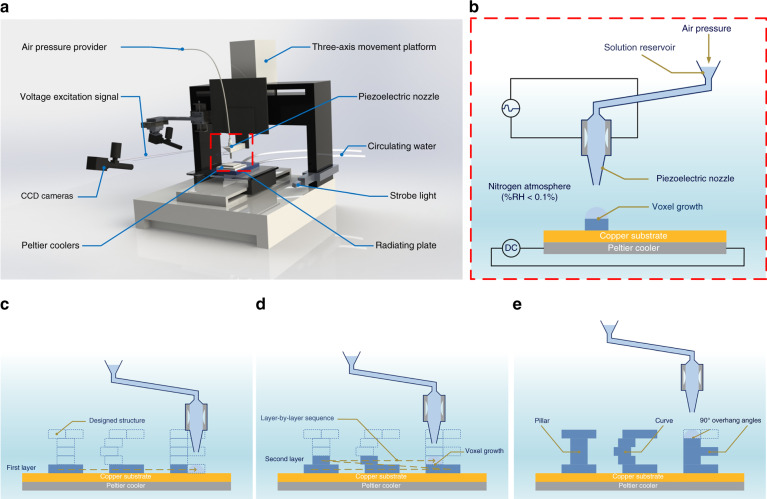
Schematic view of the principle of ice printing process. **a** Schematic view of the ice printing setup. **b** Operating principle of ice printing technology. The temperature of the substrate is controlled by Peltier coolers, which are powered with a direct current (DC) voltage source. The droplets are provided locally from the piezoelectric nozzles, thereby confining the freezing process for local ice structure patterning. The size of the droplets is controlled by the air pressure and the voltage pulse signal. **c**–**e** Layer-by-layer printing process of a 3D structure

A customized inkjet printing system has been made to demonstrate the 3D ice printing method. Figure 1a shows a schematic view of this system, which consists of a piezoelectric nozzle, a three-axis movement platform and a self-built cooling subsystem. The ice printing process is voxel based. Voxels are defined as elementary 3D blocks. A single voxel is built from the phase change of a droplet from liquid (water) to solid (ice). The size of the voxel is related to the generated droplet, which depends on the nozzle size and the applied pulse voltage signal. Stacked in a layer-by-layer sequence at defined coordinates, the voxels form the desired 2D or 3D geometry. Figure 1b demonstrates the basic working principle of ice printing. The solution is stored in a reservoir and is driven by the air pressure to fully fill the capillary in the piezoelectric nozzle. During the printing process, the droplets colliding with the ice structure spread out and freeze immediately via thermal conduction without a rebounding motion. Controlled by a computer, the modified inkjet printer can print droplets of different sizes at different frequencies in desired locations during the manufacturing process, and frozen layers are repeatedly formed with a controllable thickness and diameter, leaving ice patterns on the substrate. Theoretically, this ice printing method is suitable for a variety of substrates with good thermal conductivity.

The ice printing system can directly print 3D ice structures with micron resolution by additive manufacturing. The ice structures are flexible with respect to size, geometry and height. Figure 1c–e demonstrates the layer-by-layer printing process. The word “ICE” contains vertical structures, curved structures, and overhanging structures. After a single droplet is printed, the nozzle quickly moves to the next voxel in the same layer. The vertical distance the nozzle travels between subsequent layers is held constant throughout the printing process. The voxel is considered printed when the droplet freezes. The distance from the top of the ice structure to the bottom of the nozzle is fixed at 2 mm. Free-standing structures and overhanging angles are feasible without supporting structures.

### Effect of the experimental environment

The icing process is mainly affected by the type of solution, the temperature field above the substrate, the environmental humidity and the printing frequency.

Glass substrates are used in the following discussion. The height of the printed features had a variation of 1–3.5%, which indicates the reproducibility of the ejected droplet volume. Therefore, inkjet printing was found to be a suitable patterning technique for highly reproducible processes.

#### Solution

The formation of ice crystals is mainly influenced by the type of solution. Here, we divide the solutions into two categories. For ultrapure water, the supercooling phenomenon severely interferes with the formation of ice structures on cold substrates. As a small volume droplet is free of impurities and bubbles, even if the temperature is below 0 °C, it will remain liquid instead of freezing^27^. The supercooling phenomenon only occurs when droplets contact a clean substrate. When the droplets contact the printed ice structure, they always freeze. The supercooling phenomenon is a probabilistic event in the ice printing process because the droplets have a certain initial velocity when they hit the substrate, and the nonsteady state will help the droplets freeze. To reduce the probability of the supercooling phenomenon, an effective method is to minimize the temperature of the substrate. Related studies show that the crystallization rate of water reaches a maximum at approximately −48.15 °C, which implies the lower limit of metastability of liquid water^28^. Thanks to the two layers of Peltier coolers in the ice printing system, the current ice printing system can reach −56 °C. Another method is to modify the surface of the substrate, either introducing irregular structures to disrupt the steady state of the droplet or introducing particles to help form the ice core. For water containing impurities, its freezing point will change, which can be calculated using the following formula

1 $$\delta T = Tf^ \ast - Tf = K_fb_B = K_f\frac{{n_B}}{{m_A}}$$

When printing multiple solutions with different freezing points, selective removal of the ice structure can be achieved by adjusting the temperature of the substrate.

#### Temperature

**Fig. 2:**
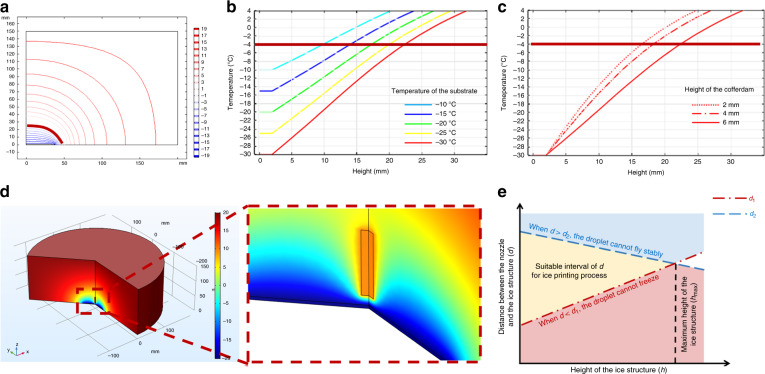
Thermodynamic simulation of the manufacturing environment in COMSOL Multiphysics; note that the red lines correspond to −4 °C. **a** Thermal field distribution of the cross section at the center of the ice structure. **b** Temperature change with respect to the height along the center of the structure for substrate temperatures ranging from −30 °C to −10 °C. **c** Temperature change with respect to the height along the center of the structure for different cofferdam heights (2, 4, and 8 mm) when the substrate temperature is −30 °C. **d** Effect of the nozzle on the thermal field distribution during printing. **e** Intervals of the distance between the nozzle and the ice structure for ice structures with different heights

The growth process of the ice crystal structure is mainly influenced by the temperature field around the substrate. Experiments have shown that ice structures should be produced at a maximum temperature of −4 °C; otherwise, the droplet freezes too slowly, destroying the intended structural design. The −4 °C isotherm height is the first reason limiting the height of the ice structure. Compared to the isolation container housing the entire printing system, the cooling system was very small. Therefore, we built a heat transfer model in an environment with an unlimited nitrogen supply. The temperature field above the substrate simulated by COMSOL Multiphysics is presented in Fig. 2a. The temperature of the bottom of the substrate was set to −10 °C to −30 °C at 5 °C intervals. As shown in Fig. 2b, as the temperature of the substrate decreased, the −4 °C isotherm height increased substantially. Cofferdams made from good thermal conductors, such as copper, can maintain the same temperature as the Peltier coolers and help control the temperature field above the substrate. When the temperature of the substrate was set to −30 °C, the height of the cofferdams on the substrate was set to 2, 4, and 8 mm. The simulation results shown in Fig. 2c reveal that a higher cofferdam helped increase the −4 °C isotherm height.

In addition, the nozzle also influenced the temperature field around itself, as shown in Fig. 2d. First, the nozzle should not be too close to the ice structure. The distance between the bottom of the nozzle and the top of the ice structure was defined as *d*. The minimum value of *d* that still allowed the droplet to freeze stably was defined as *d*_min_. As the ice height increases, the temperature of the ice surface may also increase. To reduce the thermal effect from the nozzle, the nozzle should be far from the ice, which means that *d*_min_ increases as the height of the ice structure increases. Second, the nozzle should not be too far from the ice structure. Because of the temperature gradient, the convection from the air is strong above the substrate, and a long distance will cause the flying droplet to have a random motion and deviate from the intended position. Considering the accuracy of the deposition on the substrate, a shorter distance between the nozzle and substrate will subsequently reduce the disturbance of the airflow on the flying droplet. The maximum value of *d* that still allowed the droplet to fly stably was defined as *d*_max_. Figure 2a shows that the isotherm became sparser as the height of the structure increased. In this case, the temperature changed faster and the thermal convection became more intense, which had a greater impact on the flying droplets. Therefore, *d*_max_ decreased as the height of the ice structure increased. For a stable fabrication process, *d* should always be within the bounds of *d*_min_ and *d*_max_. The available intervals of *d* for ice structures with different heights is the second reason limiting the height of the ice structure. In accordance with the simulation results and previous experimental experience, when the height of the ice structure was less than 2 mm, the distance from the nozzle to the top of the ice structure was set to 1 mm.

#### Humidity

The preservation of an ice crystal structure is mainly influenced by humidity. In a stable low-temperature environment, the main factor affecting the stability of ice is sublimation, especially for microstructures fabricated by ice printing. The sublimation rate increases substantially with decreasing humidity. However, a dry atmosphere is necessary to avoid frosting on the cold surface, which will keep the substrate clean and the surface of the ice structure smooth. Otherwise, the water molecules in the environment will form ice crystals on their surfaces. Usually, the humidity is set below 0.01% RH. To control humidity, the whole system was placed inside a container to keep it isolated from the outside. Pure nitrogen, controlled by a flow meter, was passed into the container to adjust the humidity. Although evaporation and sublimation always exist in the printing and freezing process, this setup ensured that the water molecules diffusing into the environment had less effect on humidity. In the case of continuous printing, an ultralow humidity environment can exist for at least four hours.

#### Printing frequency

The morphology of a frozen droplet is greatly affected by the printing frequency. In the discussion in this section, the nozzle did not exhibit displacement in the *x-y* plane. Hence, all of the droplets fell on the same point. The substrate was a glass slide, and the temperature of the substrate was −27 °C.

When the printing frequency was lower than 20 Hz, the freezing of each droplet was independent. As the volume of the droplet was only hundreds of picoliters, the droplet had no impact or retraction on the substrate, and it froze in less than 50 ms, which was the main difference from the macroscale behavior of the droplet^29^. Each droplet was a smooth sphere in the liquid state. When the droplet froze, because the freezing process occurred from the bottom up^30^ and the water swelled during the freezing process, the ice bead had a protrusion on the top. Although this phenomenon was inevitable, the angle of the protrusion was not affected by the rate of solidification but depended on the water density-to-ice density ratio^31^, the wetting angle^32^ and the substrate temperature^33^. For droplets of the same volume, the ice beads were always conical, so this phenomenon did not affect the resolution of ice printing technology but only limited the smoothness of microscale ice structures.

**Fig. 3:**
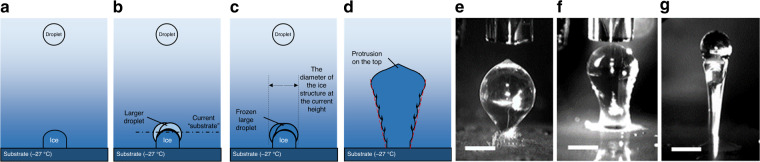
Ice torch with a large top and a small base. **a**–**d** Schematic view of the formation process. **a** The droplet freezes quickly on the cold substrate. **b** The ice structure acts as a new substrate. Droplets on the ice structure gather into a larger droplet before freezing. **c** The larger droplet freezes. The diameter of the ice structure increases as its height increases. **d** The ice torch also has a protrusion on its top. **e**–**g** Influence of printing frequency on freezing structure. The shape of the ice torch depends on the total liquid volume and the printing frequency. The printing frequencies are (**e**) 500 Hz, (**f**) 200 Hz and (**g**) 50 Hz. All scale bars are 300 μm

When the printing frequency was higher than 20 Hz and less than ~1250 Hz, the printing created torch-shaped ice structures with large tops and small bases, which was influenced by the time of the continuous printing (Videos 1–3, Supporting Information). The related results are listed in Fig. 3. Because the substrate was very cold, droplets that contacted the substrate quickly froze. For droplets that were deposited later, the existing ice structure acted as a new platform. The ice structure was not as cold as the substrate, which caused the droplets to freeze at a slightly slower rate and provided time for the droplets to accumulate in the liquid state prior to freezing. According to the abovementioned simulation results of the thermal field, the temperature around the top of the ice structure increased as the height of the ice structure increased. Therefore, the freezing rate of the droplets became slower, and the time required for the droplets to accumulate increased. Under these circumstances, the diameter of the ice structure increased with respect to the height of the structure, which led to a torch-shaped structure. Because the number of droplets on the ice structure at a certain height before freezing increased with respect to the printing frequency, the slope of the side was positively correlated with the printing frequency.

When the printing frequency is higher than 1500 Hz, the droplets are more likely to gather into a large droplet. The liquid droplets will completely cover the previously formed ice structure. In the ice printing process, the printing frequency is usually less than 1000 Hz.

Furthermore, we observed faster vertical growth in the “trigger” mode. The trigger mode is a manual operation mode in which the printing frequency *f* is very high (usually greater than 1000 Hz). Different from the continuous printing mode, in the trigger mode, the number of droplets *N* instead of the printing time *T* is set for each printing action. When *N* is far less than *f*, it seems that the printing action only produces one “large droplet” on the previous ice structure. Under this circumstance, the printing action is called a trigger. This result allows a dynamic scaling of the ice printing technique, where the size of droplets in each trigger is adjusted according to the desired feature size: depending on the size of the nozzle (20–80 μm) and the number of droplets in each trigger (1–20), we could produce voxels from 30 to 200 μm in width.

### Growth of the ice structure

**Fig. 4:**
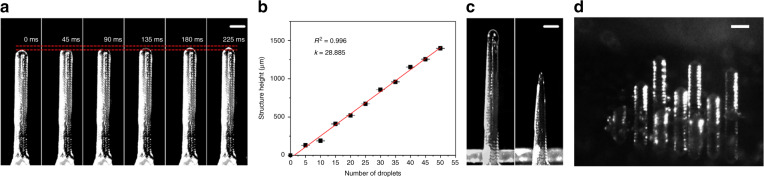
Straight ice structures fabricated by ice printing. **a** Time-sequence images showing the behavior of a single droplet deposited on a microscale ice structure; note that the time interval is 45 ms. **b** The height of the structure increased in proportion to the number of droplets. **c** A completed ice pillar and an ice needle. **d** An array of ice pillars. The heights of the ice pillars in the front line, middle line, and back line are 5 voxels, 10 voxels and 20 voxels, respectively. The scale bars are 150 μm

The growth properties of the ice crystals deposited on an ice structure were recorded using a CMOS camera with a strobe light (Video 4, Supporting Information). The ejected droplets of ultrapure water had a constant volume (~150 pL). The frequency of the droplet ejection was approximately 1 Hz. The height change of the ice structure from a single droplet is demonstrated by a set of strobe photographs from collision (0 ms) to freezing (300 ms) in Fig. 4a. The fitting curve in Fig. 4b shows that the printing process had a relatively constant layer thickness per droplet (28 ± 3 μm). As the pillar was formed by a sequence of droplet layers, the outer sidewall was not smooth, which is inevitable in the ice printing process. Moreover, because the ice crystal on the top of the pillar gradually sublimated in the ultralow humidity environment, the straight ice pillar gradually turned into a needle, as shown in Fig. 4c. Based on this phenomenon, although there were some restrictions on spatial degrees of freedom, structures smaller than the size of a single droplet could also be fabricated. Figure 4d shows an array of pillars with different heights.

**Fig. 5:**
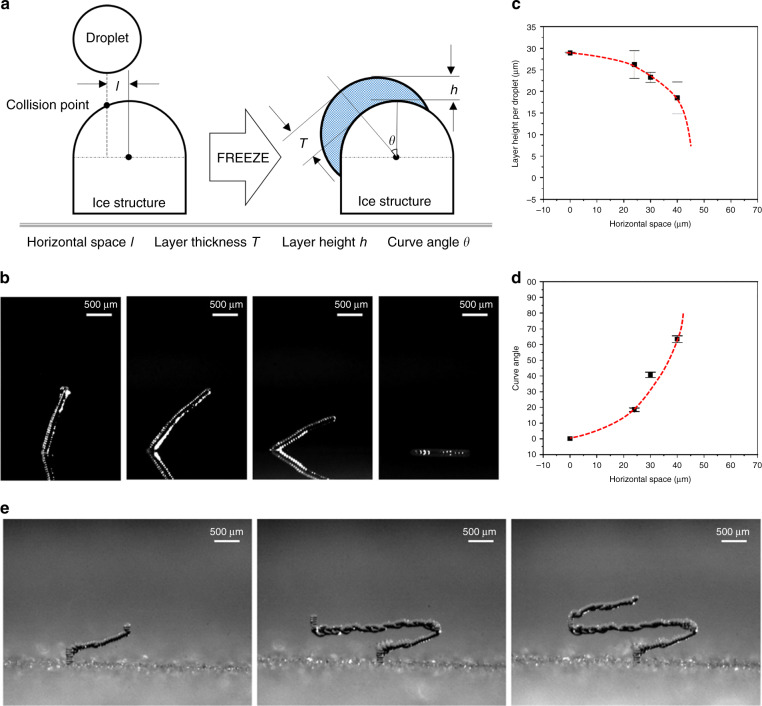
Curve structures fabricated by ice printing. **a** Mathematical model of the geometric design to estimate the relationship among the curve angle of the structure (*θ*), the horizontal distance (*l*), the layer thickness (*T*) and the layer height (*h*). **b** Ice pillars with different slopes printed when *θ* = 0, *T* = *h*, and *l* = 24 mm, 30 mm, 40 mm, and 80 mm. **c** Relationship between *l* and *h*. **d** Relationship between *l* and *q*. **e** The fabrication process of a horizontal beam

Pillars with different slopes can be achieved by controlling the horizontal space between printed droplets, as illustrated in Fig. 5a. When printing automatically, the horizontal space *l* is related to the ratio of the nozzle velocity *v* and the printing frequency *f* as follows:

2 $$l = \frac{v}{f}$$

In most instances, *l* should be smaller than the radius of the frozen droplet; otherwise, the ice pillar will be discontinuous rather than the designed shape. In Fig. 5b, the velocity of the nozzle is fixed at 6 mm/s, and the printing frequencies—from left to right—are 125, 100, 75, and 50 Hz. Note that *l* affected both the layer height *h* (Fig. 5c) and the curve angle *θ* (Fig. 5d).

In addition, overhanging structures with 90° angles or even negative angles can be fabricated by manual control. In the manual control mode, the landing point of a droplet is predicted through the charge-coupled device (CCD) camera. When printing each droplet, we managed to make the droplets, which flew obliquely downward, contact the side of the ice structure. In this way, the droplet will continue moving downward along the surface of the ice structure instead of staying steadily on the top of the ice structure. Because the volume of the droplet is very small, it will freeze and become an extension of the existing ice structure before falling off of the ice structure. In this way, horizontal growth and diagonal downward growth of the ice structure are achieved. Figure 5e demonstrates the growth process of a horizontal structure.

### Printing results

Figure S1 illustrates the printing process and the printing results of flat graphics. Arbitrary patterns were created by exploiting the *x-y* positioning capabilities of the movement platform (Video 5, Supporting Information).

**Fig. 6:**
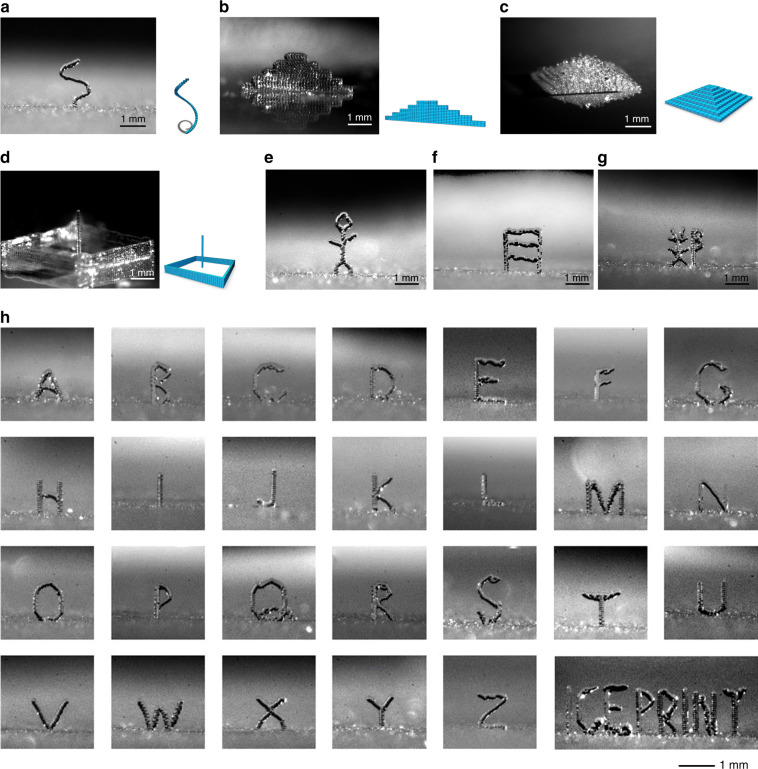
Images of 3D-printed ice structures at the microscale. **a** A single helical ice structure. **b** An ice wall with various heights. **c** An ice pyramid. **d** A composite ice structure. **e** An ice stickman. **f** An ice ladder. **g** A Chinese character (zheng). **h** An ice alphabet including various 3D overhanging structures without support

A range of 3D structures is displayed in Fig. 6. Figure 6a is a helix on one voxel in diameter; the voxels are stacked in one dimension. Figure 6b is a wall structure with different heights from 5 voxels to 25 voxels and a wall thickness of 1 voxel; the voxels are stacked in 2 dimensions. Figure 6c is a pyramid where the voxels have been stacked in 3 dimensions. Figure 6d shows a combination of four connecting walls and a pillar in its center, which were all printed in one run. Figure 6e–g shows a stickman, a three-story ladder and a Chinese character (zheng). A microscale ice alphabet is shown in Fig. 6f. The reproducibility of the printing structure is shown in Fig. S3 (Supporting Information).

## Discussion

Utilizing the self-built printing system, precise control of various microscale ice structures was achieved. However, as icing is a complicated physical process, the shape of the frozen droplet and its position on the ice structure are not always as expected. There are still few limitations in ice printing technology.

One possible limitation of ice printing is the growth characteristics of ice crystals. Droplets always freeze from where the ice core first forms. As previously mentioned, the ice voxel is an irregular hemisphere with a protrusion on its top. The protrusion provides disturbance to the droplet, which increases the probability of the ice cores forming here first. When local growth is initiated, the generated protrusion will localize the growth of the ice structure. Although the wall in Fig. 6b was printed horizontally in a sequence of lines, the wall contains grooves and appears as vertically aligned pillars.

Another possible limitation is that the voxel-based fabrication method makes the outer surface of the ice structure rough, especially when machining bevel structures. A close inspection of the pyramid in Fig. 6c reveals an obviously stepped bevel. For printing results without overhanging structures, we can slowly increase the substrate temperature to −4 °C, and there will be a quasi-liquid layer on the solid–vapor interface of the ice structure. The thin water film adheres tightly onto the ice structure and will by smoothed by its surface tension. When the ice structure freezes again at a low temperature, the thin water film will form a relatively smooth outer surface.

A third possible limitation is the instability of the icing process, especially when printing structures with nearly 90° angles or even negative angles. Taking the ladder in Fig. 6f as an example, the three horizontal lines were all printed from left to right, but the final shapes are not exactly the same. Assuming that the printing state of the nozzle is stable, the air flow caused by the uneven temperature field will affect the flight path and the subsequent landing point of the droplet. When the droplets flew obliquely at different angles and hit the side of the previous structure, the ice structures had different protrusion orientations, thereby affecting the next icing process.

## Conclusion

In conclusion, we have demonstrated ice printing technology as a 3D fabrication method, which represents a new step in the direction of using inkjet printing technology for the microfabrication of ice structures. Ice patterns with defined geometries were created with a resolution in the range of tens of micrometers by providing the desired droplets on the cold substrate. Using different voltage excitation signals, the spot sizes can be tuned. In addition, as nanowires have been successfully assembled in macroscale ice structures^34,35^, microscale ice structures can potentially support 3D porous scaffolds for materials that are soluble or dispersed in a liquid of metal salt nanoparticles, which precipitate from the water during the freezing process and then occupy the spaces between ice grains. We plan to explore the suitability of our setup to enable the fabrication and preservation of complex three-dimensional ice structures with different materials. Furthermore, the produced microscale ice structures can be used as a mold for complex microfluidics with presealed liquids, which is difficult or even infeasible for conventional photolithography methods. In the future, this protocol can potentially enable biomicrofluidic devices, such as drug mixing and delivery systems.

## Methods

### Printing system

A custom inkjet printing system was constructed to demonstrate the 3D ice printing method. Figure S2 shows a photograph of the system, which consists of a piezoelectric nozzle, a three-axis movement platform and a self-built cooling subsystem. The Piezoelectric nozzles, air pressure controller and voltage signal generator were purchased from MicroFab Technology, USA. The inside diameters of the piezoelectric nozzles ranged from 2 to 60 μm, which could meet the printing needs of solutions with different viscosities and compositions. The ranges of the three-axis movement platform in the *x*, *y* and *z* axes were 300, 150 and 40 mm, respectively. All three stages had a 10 μm resolution, which satisfied the requirements of microfabrication. The platform could be manually controlled with a handle or automatically controlled with a computer program. The signal coupling of the piezoelectric nozzle and the movement platform was achieved through an I/O controller (ZLAN 6802) connected with a computer.

A cold surface was required for the working platform on which freezing occurs. The cooling system (which could reach a minimum temperature of −56 °C) consisted of two Peltier coolers and a radiating plate with circulating water at 5 °C. The DC voltage source controlled the heat transfer from the cold face to the hot face of the Peltier coolers. The radiating plate, which was connected to a water-cooling system, was used to release the heat from the hot side of the Peltier coolers. These two parts had a uniform layer of silver-containing thermal grease between them to enhance heat transfer. A platinum thermocouple was used to measure the working temperature of the cold surface in the cooling system. All the self-built cooling systems were surrounded by foam insulation to isolate the heat exchange with the environment.

During the printing process, the droplets collided with the ice structure and then spread out and immediately froze via heat conduction without a rebounding motion. With a certain nozzle size and voltage signal, frozen layers were repeatedly formed with a constant thickness and diameter, leaving ice patterns on the substrate. Controlled by a computer, the modified inkjet printer can print droplets of different sizes at different frequencies in the desired location during the manufacturing process.

The whole ice printing system was placed in a container with a nitrogen atmosphere to isolate the system from the outer environment. Icing is a complex physical process during which humidity and temperature are the main factors. Hence, a digital thermometer and hygrometer (Sensirion, Switzerland) were used to monitor the environment inside the container.

The system is flexible, enabling the user to design custom deposition processes. For example, here, we added two CCD cameras (front and side) to enable visualization of the nozzle loading, the generated droplets and the printed structures.

When manually controlled, the printing process was monitored by the two CCD cameras. The landing point of the droplet can be confirmed by observing the position on the screen. When the printing process was automated by a preset program, the trajectory, the movement speed and the voltage control of the nozzle were specified in a.*tsp* file. The file was loaded into the printer software. The.*tsp* files were generated by a design assistant called TSAPS, which was provided by Shenteng Technology, China. Alternatively, the files can be generated by any third-party software capable of exporting CAD files.

## Supplementary information

Video 1

Video 2

Video 3

Video 4

Supporting Information File
