# Supplementary material for: Inkjet printing-based fabrication of microscale 3D ice structures
Source: Microsyst Nanoeng. 2020 Oct 19;6:89. doi: 10.1038/s41378-020-00199-x (PMC8433306; doi:10.1038/s41378-020-00199-x)
Supplement: Supplementary file 5 — Supporting Information File [file 41378_2020_199_MOESM5_ESM.docx]

Supporting Information

**INKJET PRINTING-BASED FABRICATION OF MICROSCALE 3D ICE STRUCTURES**

**Fengyi Zheng**^1^**, Zhongyan Wang**^1^**, Jiasheng Huang**^1^**, and Zhihong Li**^1,*^

^1^National Key Laboratory of Science and Technology on Micro/Nano Fabrication, Institute of Microelectronics, Peking University, Beijing 100871, CHINA

^*^Corresponding author: zhhli@pku.edu.cn

Fabrication capabilities for flat graphics


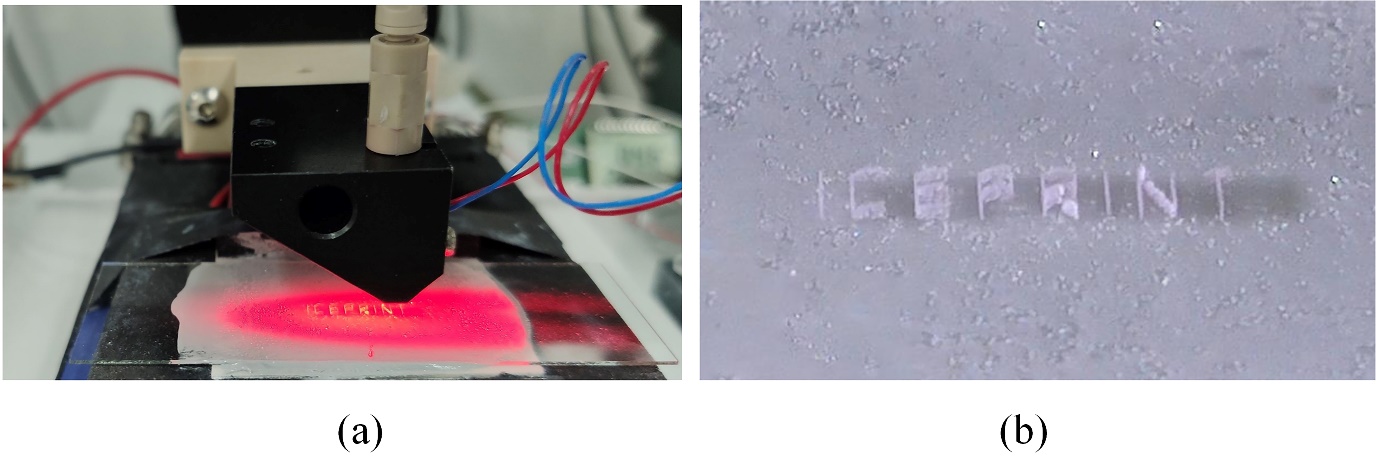


**Figure S1.** Images of 3D printed ice structures at the microscale. (a) Ice patterns in printing process. (b) Printing result of flat graphics "ICEPRINT". The width of the text is about 120 μm and the height of the text is about 1.5 mm.

Setup for ice printing system


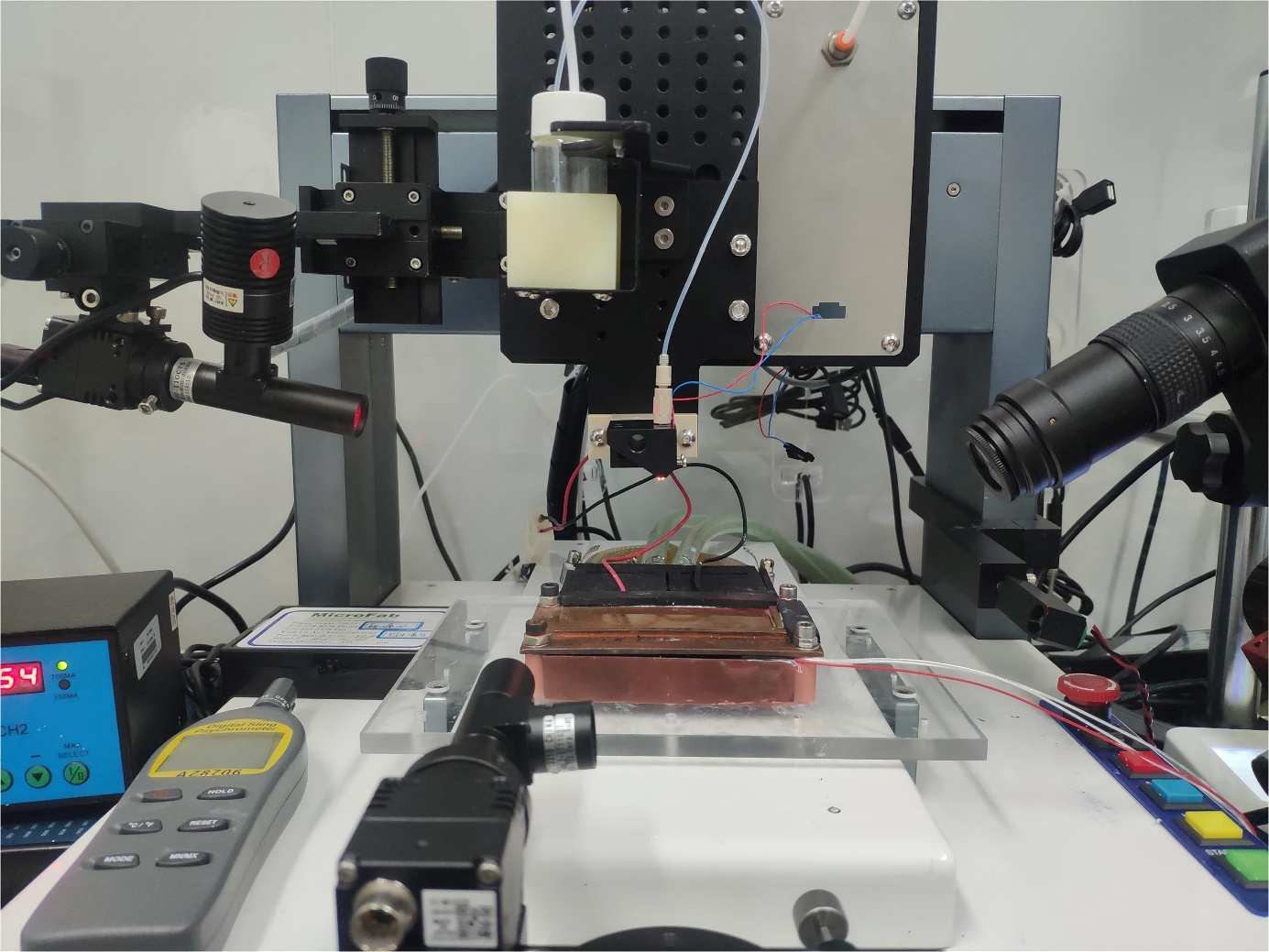


**Figure S2.** Image of the ice-printing system.

Reproducibility of ice structures


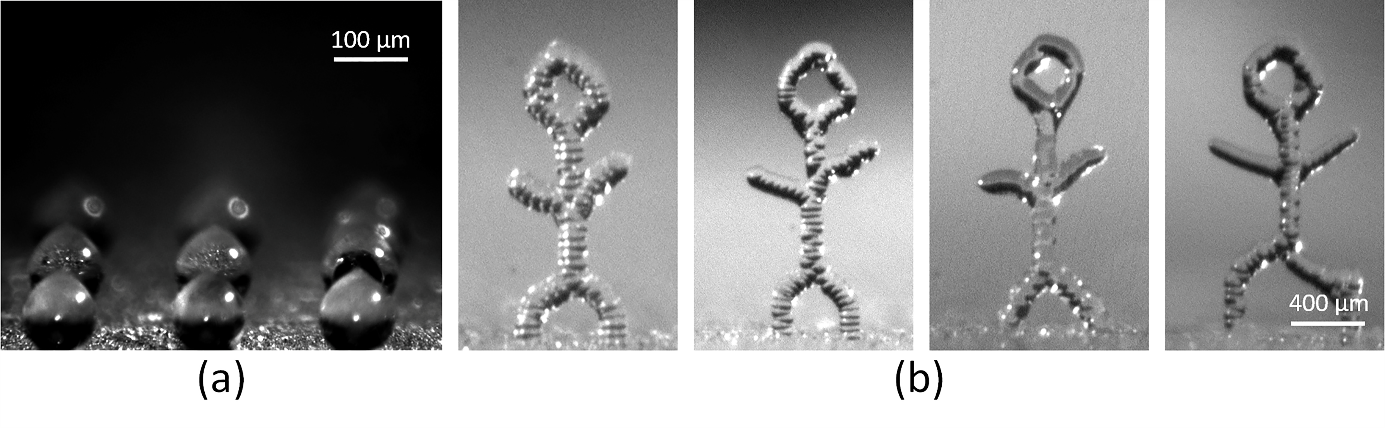


**Figure S3.** (a) Image of ice beads array, fabricated in automatic mode. All the beads have the same shape. Ice structures with same program have good reproducibility. (b) Images of ice stickman, fabricated in manual mode. Compared with automatic mode, manual mode has slight lack of reproducibility, but can still roughly maintain the same shape.
